# Vaginal Inflammatory Status in Pregnant Women with Normal and Pathogenic Microbiota in Lower Genital Tract

**DOI:** 10.5402/2011/835926

**Published:** 2011-04-26

**Authors:** Sebastián Galiñanes, Enrique Coppolillo, Maximiliano Cifarelli, Martha Cora Eliseht, Ercilia Pellisa, Mirta Losada, Sebastián Gruccio, Hilda Ruda Vega, Carlos Vay, Angela Famiglietti, Beatriz Perazzi

**Affiliations:** ^1^Clinical Bacteriology Laboratory, Department of Clinical Biochemistry, Hospital de Clínicas, Faculty of Pharmacy and Biochemistry, University of Buenos Aires, Córdoba 2351, 1120, City of Buenos Aires, Argentina; ^2^Lower Genital Tract Clinic, Obstetrics Division, Department of Tocogynecology, Hospital de Clínicas, University of Buenos Aires, Córdoba 2351, 1120, City of Buenos Aires, Argentina

## Abstract

*Objective*. To assess the vaginal inflammatory status (VIS) in pregnant women, whether symptomatic or asymptomatic, by leukocyte quantification in relation to the microbiota during each pregnancy trimester (T). *Materials and Methods*. A thousand two hundred and forty eight vaginal exudates from pregnant women were prospectively examined. All the patients underwent a clinical and colposcopic examination and a microbiological study of vaginal exudates. Leukocyte quantification was determined by May-Grunwald Giemsa staining as LNR per field (400X). *Results*. Statistically significant differences (SSD) in LNR were observed in the VIS of asymptomatic patients (AP) compared with that of symptomatic ones (SP) with normal microbiota: 10–15 for the 1st T, <10, 20 to 25 and >25 for the 2nd T and >25 for the 3rd; with candidiasis: <10 for the 1st T, <10, 15 to 20 and >25 for the 2nd T and <10 and >25 for the 3rd T. In women with trichomoniasis, SSD in the LNR were observed between SP with LNR ≥ 10 and AP with NLR < 10 in the three trimesters altogether. In women with BV, no SSD were observed in the LNR of any AP with respect to SP for the three T. *Conclusion*. The VIS is influenced by vaginal microbiota and depends on the state of pregnancy and also, on gestational age. The pronounced leukocyte increase in asymptomatic patients in the absence of lower genital tract infection during the third trimester of pregnancy should be highlighted.

## 1. Introduction

Several hormonal changes are produced during pregnancy that can increasingly predispose to infections of the lower genital tract [1–4].

These infections are associated with a great number of gynecologic and obstetric complications, such as preterm birth [5–13], premature rupture of the membranes [14–16], chorioamnionitis [17, 18], postpartum endometritis [8], inflammatory pelvic disease [19, 20], intrauterine growth retardation [21], and low birth weight [13].

These maternal and perinatological complications could be partly triggered by the local immune response as part of the pathogenic mechanism generated by the infection [22].

However, it is essential to ascertain whether this immunological response of the vaginal mucosa is actually influenced by gestation. There is no literature available referring to the vaginal inflammatory status (VIS) during pregnancy expressed as leukocyte numerical range (LNR) by microscopic observation.

The aim of this work was to assess VIS in pregnant women, whether symptomatic or asymptomatic, by leukocyte quantification in relation to the normal microbiota and the presence of candidiasis, trichomoniasis, and bacterial vaginosis (BV) during each trimester of pregnancy (T).

## 2. Materials and Methods

A thousand two hundred and forty-eight vaginal exudates from pregnant women (231, 359, and, 658 in the first, second and third T, resp.) who were consecutively and prospectively examined at the Obstetrics Clinic at Hospital de Clínicas of the University of Buenos Aires, Argentina, from July 1, 2005 to December 31, 2008, were analyzed. This study was approved by the Hospital Ethics Committee. All the patients underwent a clinical and colposcopic examination and a microbiological study of vaginal exudates. Symptomatic patients showed pruritus and/or an increase in vaginal discharge, which was thick, sticky, purulent, smelly, thin and/or homogeneous depending on the case. The microbiological study of vaginal exudates included the following examinations:

- smears for Gram and prolonged May-Grunwald Giemsa staining,
- microscopic wet smear examination with 1 mL of physiologic saline solution,
- microscopic wet smear examination with sodium-acetate acetic-acid formalin (SAF)/methylene blue (0.5 mL methylene blue and 0.5 mL SAF) [23],
- pH determination of vaginal exudates,
- fishy-odor test with 1 mL of 10% KOH with posterior microscopic wet smear examination,
- liquid medium culture (modified tioglycolate medium) for *Trichomonas vaginalis* detection with seven-day incubation period at 37°C in an atmosphere of 5% CO_2_ [24], and
- solid medium culture (Modified Columbia Agar) in 5% human blood plates with 48 hour incubation period at 37°C in an atmosphere of 5% CO_2_. The sample was preserved in Stuart transport medium.

The presence of Gram-positive bacilli in the Gram staining and the development of small, point-like, and *ά*-hemolytic colonies in the solid medium culture (Modified Columbia Agar) in 5% human blood plates, was considered normal or lactobacillary microbiota.

Candidiasis detection was performed by microscopic wet smear examination with 1 mL of 10% KOH and by Sabouraud and blood agar culture. 

The diagnosis of bacterial vaginosis (BV) was performed by means of the Nugent's method through the determination of a score ≥7 in the Gram stain [25], and by the Amsel's method, that is the presence of three or more of the following criteria [26]:

- “clue-cells” in the Gram stain,
- pH ≥ 4.5,
- positive fishy-odor test, and
- thin and homogeneous vaginal discharge.

*T. vaginalis* detection was performed by direct microscopic examination with physiologic saline solution and SAF/methylene blue, prolonged May-Grunwald Giemsa staining, and modified thioglycolate medium, which was examined daily by wet smears for the detection of motile parasites.

Leukocyte quantification (without clumping) was determined by May-Grunwald Giemsa staining and was expressed as LNR per field (400x), by observation of 10 nonadjacent microscopic fields.

### 2.1. Exclusion Criteria

Patients showing cervicitis in the colposcopic exam were not included in the study.

### 2.2. Statistical Analysis

Fisher's and chi-square tests *χ* ^2^ were performed to assess the VIS in relation to microbiota, applying the calculation of adjusted residual (AR) in those cases with *P* ≤ .05 to identify the LNRs which yielded the significance levels obtained (Epi info 6.04). LNRs with AR of ≥±2 were considered those yielding the significance levels obtained.

## 3. Results

Statistically significant differences resulting from the LNR were observed in the VIS of asymptomatic pregnant patients with normal microbiota compared with that of symptomatic ones: 10 to 15 for the first trimester, <10, 20, to 25 and >25 for the second trimester, and >25 for the third trimester (*χ* ^2^: 10.73, *P:*.03, AR: ±2.32; *χ* ^2^: 11.11, *P*:  .02, AR: ±2.32 ± 2.02 y ± 2.50; *χ* ^2^: 10.53, *P*:  .03, AR: ±3.04, resp.) (Table 1).

In those patients with candidiasis, statistically significant differences in the LNR were observed in the VIS of asymptomatic patients compared with symptomatic ones: <10 for the first trimester, <10, 15 to 20, and >25 for the second trimester, and <10 and >25 for the third trimester (*χ* ^2^: 10.40, *P*:  .03, AR: ±2.63; *χ* ^2^: 17.60, *P*:  .00, AR: ±2.65 ± 2.58 y ± 2.14; *χ* ^2^: 14.24, *P*:  .00, AR: ±3.47 and ±2.09, resp.) (Table 2).

With regard to patients with trichomoniasis (*n*: 38), statistically significant differences in the LNR were observed between symptomatic patients with LRN ≥ 10 and asymptomatic patients with LNR < 10 in the three pregnancy trimesters altogether (*P* Fisher:  .01) (Table 3).

In women with BV, no statistically significant differences were observed in LNR of asymptomatic patients with respect to symptomatic ones in any of the three trimesters (*χ* ^2^: 7.05, *P*:  .13; *χ* ^2^: 3.86, *P*:  .43; *χ* ^2^: 5.17, *P*:  .27, resp.) (Table 4).

## 4. Discussion

With advancing gestation, a gradual increase in LNR (first trimester: 10 to 15, second trimester: 20 to >25, and third trimester: >25) was observed in pregnant women with lactobacillary microbiota. Therefore, in the last pregnancy trimester, the >25 LNR was statistically significant in the absence of lower genital tract infection, mainly in asymptomatic patients. Other authors such as Yamada et al. [27] also described an increase in the number of leukocytes and interleukin 8 (IL-8) as gestation advanced. 

Furthermore, Gilbert et al. [28] reported that a significant increase of proinflammatory cytokines (IL-6 and IL-8) is produced in the third trimester. These cytokines act as chemoattractants of polymorphonuclear leukocytes stimulating the expression of prostaglandins in response to delivery preparation. Thus, these interleukins may be considered markers for subsequent normal or preterm labor [29]. 

In addition, Gilbert et al. [28] observed that a decrease in the production of inflammatory cytokines may occur during the second trimester of pregnancy as part of the normal course of pregnancy. Therefore, an increase of those cytokines in this trimester would produce preterm labor probably related to an intrauterine or lower genital tract infection. 

Furthermore, Gilbert et al. [28] reported that even in the case of intrauterine bacterial infection, the increase of inflammatory cytokines is more pronounced than that expected in a normal pregnancy in response to labor preparation.

According to the results of this study, the patients with symptomatic candidiasis developed colpitis and were associated with LNR greater than 25 in the second and third trimester with positive microscopic examinations, whereas the colposcopy was normal in asymptomatic candidiasis, showing LNR lower than 10 in the three pregnancy trimesters with generally negative microscopic examinations. Furthermore, the patients with symptomatic trichomoniasis developed colpitis and were associated with LNR greater than 10, with positive microscopic examinations. On the other hand, in patients with asymptomatic trichomoniasis, the colposcopy was normal and was only detected by culture, that is, with generally negative microscopic examinations and showing LNR under 10, considering the three trimesters altogether.

However, no statistically significant differences were observed in any of the LNR of either symptomatic or asymptomatic patients with BV, since this infection usually occurs in the absence of an inflammatory reaction. 

These results agree with those described in the literature referring to the association of proinflammatory interleukins (IL-6 and IL-8) with vaginitis, whereas in BV, there is an increase of IL-1*β* (though lower than in vaginitis) but not of the interleukins mentioned above [29, 30]. In addition, Nenadić et al. observed a correlation between leukocyte count and IL-8 concentration and proposed microscopic observation of the number of leukocytes as a useful tool for identifying of patients with vaginal inflammation [31].

It is worth noticing that this work is the first report in the literature referring to vaginal inflammatory status during pregnancy expressed as leukocyte numerical range by microscopic observation, a universally studied methodology used for the diagnosis of infections of the lower genital tract in primary health care.

## 5. Conclusion

The vaginal inflammatory status is influenced by vaginal microbiota and does not only depend on the state of pregnancy but also on gestational age. Therefore, the pronounced leukocyte increase in asymptomatic patients in the absence of lower genital tract infection during the third trimester of pregnancy should be highlighted.

## Figures and Tables

**Table 1:** Distribution per trimester of leukocyte numerical ranges in pregnant patients, whether symptomatic or asymptomatic, with normal microbiota.

	1st trimester l/f^1^					2nd trimester l/f^1^					3rd trimester l/f^1^				
	<10	10–15	15–20	20–25	>25	<10	10–15	15–20	20–25	>25	<10	10–15	15–20	20–25	>25
Asymptomatic	91	11	6	4	7	157	15	11	6	9	307	45	11	19	29
Symptomatic	7	4	0	2	0	8	1	1	2	3	11	2	1	0	5
AR^2^	1.77	**−2.32**	0.83	−1.98	0.90	**2.32**	0.13	−0.18	**−2.02**	**−2.50**	1.63	0.06	−0.67	0.96	**−3.04**
	−1.77	**2.32**	−0.83	1.98	−0.90	**−2.32**	−0.13	0.18	**2.02**	**2.50**	−1.63	−0.06	0.67	−0.96	**3.04**

^1^Leukocytes/field.

^2^Adjusted residuals.

**Table 2:** Distribution per trimester of leukocyte numerical ranges in pregnant patients, whether symptomatic or asymptomatic, with candidiasis.

	1st Trimester l/f^1^					2nd Trimester l/f^1^					3rd Trimester l/f^1^				
	<10	10–15	15–20	20–25	>25	<10	10–15	15–20	20–25	>25	<10	10–15	15–20	20–25	>25
Asymptomatic	25	3	0	0	1	27	7	1	1	2	52	8	2	2	7
Symptomatic	8	2	2	2	2	17	2	9	4	9	26	8	7	5	14
AR^2^	**2.63**	−0.22	−1.95	−1.95	−1.17	**2.65**	1.89	**−2.58**	−1.30	**−2.14**	**3.47**	−0.36	−2.00	−1.40	**−2.09**
	**−2.63**	0.22	1.95	1.95	1.17	**−2.65**	−1.89	**2.58**	1.30	**2.14**	**−3.47**	0.36	2.00	1.40	**2.09**

^1^Leukocytes/field.

^2^Adjusted residuals.

**Table 3:** Distribution in the three trimesters of leukocyte numerical ranges in pregnant patients, whether symptomatic or asymptomatic, with trichomoniasis.

	L/f	
	<10	10–>15
Asymptomatic	12	4
Symptomatic	7	15

L/f: Leukocytes per field.

**Table 4:** Distribution per trimester of leukocyte numerical ranges in pregnant patients, whether symptomatic or asymptomatic, with bacterial vaginosis.

	1st trimester l/f^1^					2nd trimester l/f^1^					3rd trimester l/f^1^				
	<10	10–15	15–20	20–25	>25	<10	10–15	15–20	20–25	>25	<10	10–15	15–20	20–25	>25
Asymptomatic	19	1	0	0	0	13	3	2	0	1	29	8	3	0	5
Symptomatic	23	5	1	2	5	34	5	12	5	6	39	5	1	2	9

^1^Leukocytes/field.
